# Efficacy of oral contrast agents for upper gastrointestinal signal suppression in MRCP: A systematic review of the literature

**DOI:** 10.1177/2058460117727315

**Published:** 2017-08-30

**Authors:** Anne Frisch, Thula C Walter, Bernd Hamm, Timm Denecke

**Affiliations:** 14903 Charité – Universitätsmedizin Berlin, corporate member of Freie Universität Berlin, Humboldt-Universität zu Berlin, and Berlin Institute of Health, Department of Radiology, Germany

**Keywords:** Magnetic resonance imaging (MRI), bile ducts, contrast media, magnetic resonance cholangiopancreatography (MRCP), pancreatic ducts, literature review

## Abstract

**Background:**

Orally administered substances which suppress signals from gastrointestinal fluid can be used to enhance image quality in magnetic resonance cholangiopancreatography (MRCP). In daily practice, the available substances range from commercial products to regular viands such as fruit juices.

**Purpose:**

To provide an overview on the significance of and the substances used as gastrointestinal fluid signal suppressors in MRCP.

**Material and Methods:**

A systematic review of the existing literature was performed to evaluate the efficacy and efficiency of oral T2-signal suppressors in MRCP.

**Results:**

Twenty-five publications on 16 different oral contrast media were identified. The most commonly used substances were ferumoxsil, ferric ammonium citrate, and pineapple juice. Twenty-three out of 25 publications supported the use of oral signal suppressors in MRCP. Advantages of oral signal suppressors include improved visualization of the pancreatobiliary ductal system, increased help with differential diagnoses, and higher detection rates of relevant diagnoses due to a reduction of overlaying signals.

**Conclusion:**

The application of oral substances for gastrointestinal signal suppression in MRCP is recommendable. A variety of substances are used in daily routine with good but varying effectivity and patient tolerance.

## Introduction

**Fig. 1.**
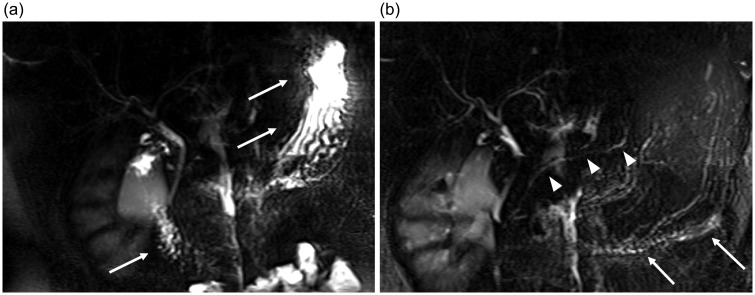
Normal findings of a single shot thick slab MRCP obtained in the same healthy volunteer in one examination protocol, (a) without and (b) with pineapple juice (100% NFC juice [not from concentrate], Voelkel, Germany). (a) Without contrast agent, high intensity signals within the GI tract (arrows) lead to superimposition and impaired assessability of the pancreatic duct. (b) GI signals are reduced (arrows) and the pancreatic duct is revealed (arrowheads).

**Fig. 2.**
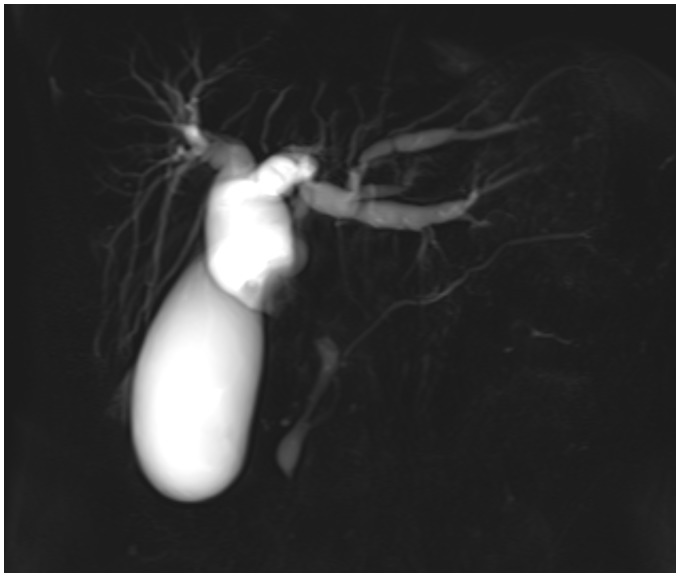
Single shot thick slab MRCP of a patient with an extrahepatic tumor, obtained with an orally administered, signal suppressing contrast material (ferumoxsil, Lumirem®, Guerbet, France). An evenly suppression of high intensity signals caused by fluids in stomach and duodenum was achieved. The extrahepatic lesion leads to a subtotal stenosis of the common hepatic duct (seen as lacking hyperintense signal) and a consecutive dilatation of intrahepatic bile ducts. The pancreatic duct is not affected.

**Fig. 3.**
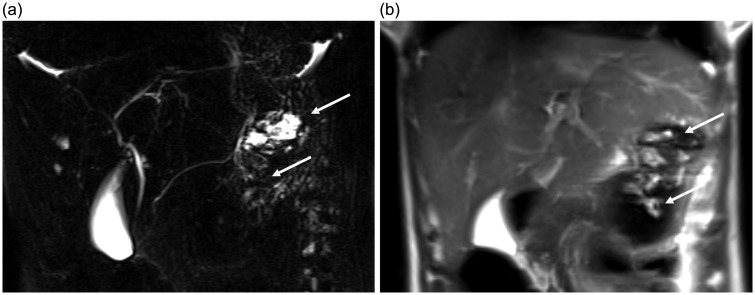
(a) Single shot thick slab MRCP after the oral administration of signal suppressing contrast material (ferumoxsil, Lumirem®, Guerbet, France) within the diagnostic investigation of recurrent episodes of pancreatitis. High intensity signals within the region of the pancreas tail (arrows) lead to an impaired assessability of the pancreatic duct. (b) The correlation to coronal T2W HASTE MR confirm the stomach as origin of superimposing signals (arrows).

Magnetic resonance cholangiopancreatography (MRCP) is the non-invasive imaging technique of choice to evaluate the pancreatobiliary system. Fluid in the biliary and pancreatic ducts is visualized (Fig. 1) with heavily T2-weighted (T2W) sequences (1–4). These images permit a detailed assessment of the anatomy and possible pathology of the duct system and the neighboring organs, which is of increasing relevance, as many pancreatobiliary pathologies such as biliary obstruction (Fig. 2) and intraductal papillary mucinous neoplasm (IPMN) (5,6) are assessed and followed up with magnetic resonance imaging (MRI) and MRCP. However, even in fasting patients, superimposed high signal intensity of fluids in the upper gastrointestinal (GI) tract (e.g. saliva, bile, or pancreatic secretions) can greatly hamper diagnostic image quality (Fig. 3) (7). To avoid this problem in advance, a reduction—or better yet, an elimination—of fluid signal from the upper GI tract is desirable. Apart from fasting prior to the examination, orally administered (negative) contrast agents with a strong T2 shortening effect allow a suppression of high intensity fluid signal and are commonly used for patient preparation (8,9).

The purpose of this study was to provide an overview on the substances used as GI fluid signal suppressors in MRCP and their clinical efficacy and efficiency.

## Material and Methods

A systematic review of the existing literature was performed in PubMed (https://www.ncbi.nlm.nih.gov/pubmed/). The search terms listed in Table 1 were used in 16 different combinations. The search covered publication dates from 1990 to 2015. The resulting publications were categorized as original articles or case reports and if they were available as full text. All articles on oral signal suppressors in MRCP were analyzed regarding their recommendation of an oral GI signal suppressor and the type of agent used. Advantages and disadvantages of oral signal suppressors and their use in MRCP were also noted.

**Table 1.** Results of the literature research.

Sequence of search item combinations	Publications found (n)	Evaluable publications (n)
Cholangiopancreatography + MR + contrast agent	58	20
Cholangiopancreatography + MR + oral gastrointestinal contrast agent	11	0
Cholangiopancreatography + MR imaging + contrast agent + oral	22	0
MRCP + oral gastrointestinal contrast agent	10	0
MR + biliary system + oral gastrointestinal contrast agent	1	0
MR + biliary system + contrast agent	29	0
Manganese chloride tetrahydrate + MRCP	3	1
Manganese chloride tetrahydrate + MRI	8	0
Ferumoxsil + MRCP	8	1
Ferumoxsil + MRI	39	2
MRCP + negative + contrast	61	1
Lumirem + MRCP	9	0
Gastromark + MRCP	8	0
Lumenhance + MRCP	0	0
Ferristene + MRCP	1	0
Lumivision	0	0
	268	25

Results of the literature research arranged according to search items and amount found as well as further analyzed publications in chronologic order of the search. Repeatedly found and as useful categorized works were not mentioned among the used publications in the following lines.

## Results

A total of 268 publications were identified in the systematic literature review. Of these, 25 publications met the inclusion criteria and were selected for further analysis. In these 25 studies, 16 different orally administered contrast agents were used to alter the GI fluid signal in MRCP. Twelve of the 25 publications covered fruit juices and similar beverages, 18 articles studied certified medical products. A list of all identified substances (including chemical name, trade name, distributing company) and the agents they were compared to are listed in Supplementary Table 1 (online).

The administration protocols differed immensely between publications. Eleven articles did not provide information on patient preparation. The shortest fasting period provided was 3 h (10), the longest was 12 h (11) with a median 6 h (12–16). The administered amount of contrast agent ranged from 10 mL (17) to 1000 mL (18); two articles did not supply any information on the administered amount of oral contrast (19,20). In most cases (n = 11), MRCP was performed immediately after the oral ingestion (11,17–27). In all other cases (n = 14), time intervals between oral administration and the beginning of the scan ranged between 5 min (10,28,29) and 180 min (20). Four articles evaluated more than one time point after the oral ingestion of black tea, pineapple juice, or manganese chloride tetrahydrate (MCT; Bothdel Oral Solution®, Kyowa Hakko Kirin, Chiyoda, Tokyo, Japan or LumenHance®, Bracco Diagnostics, Milan, Italy), respectively (15,20,28,29).

The majority of publications (n = 18) studied MRCP protocols with and without the use of signal suppressors (certified products [n = 9] (9,10,16–18,21,25,26,30), fruit juices/beverages [n = 7] (11,13–15,21,28,29), pineapple juice in combination with non-absorbable gadolinium-chelate [n = 2] (22,24); Supplementary Table 1). Four compared certified agents to other products (fruit juices/beverages [n = 2] (21,31) or other certified agents [n = 2] (26,32)). One publication analyzed image quality at different time points after the intake of a single certified product (MCT; Bothdel Oral Solution®) (20).

Six publications were observatory nature in regard to the effect of the used signal suppressor (n = 5 on certified products, n = 1 on fruit juices). These were either case reports, descriptions of experiences without a structured scientific approach, or publications with a different study focus (i.e. detection of duodenal diverticula) (12,18,19,23,27,33). For example, Delaney et al. evaluated safety, feasibility, and accuracy of MRCP in children. The study included an assessment of the visualization and size of the pancreatic duct before and after the intravenous application of secretin. However, the impact of secreted pancreatic fluid on the intraduodenal signal suppressing agent was neither assessed nor discussed (23).

Twenty-two publications studied MRCP in adult patients (9–14,16–33). Two studies investigated MRCP in pediatric patients (12,23). Five studies were performed in volunteers (11,15,20,28,32). A qualitative assessment of the obtained images was done in 19 articles and covered a wide range of evaluated characteristics. The majority of articles assessed the impact on the depiction of particular pancreatobiliary ductal structures (9,11,13–15,20,21,24–26,28–31). Some studies focused on the overall image quality of the pancreatobiliary ductal system with (10,13,15,22,26,28,32) or without (21) accounting for overlaying GI fluid signal. Four publications evaluated the pure effect of the agent on suppressing GI fluid signal (21,29,31,34). Three studies included a comparison between pre- and post-contrast MRCP to assess the impact on overall image quality (9,10) or the impact on the diagnosis, respectively (11). Mostly, Likert scales were used for the qualitative assessments, covering 3-, 4-, 5-, and 7-point-scales. Three out of 25 publications used dichotomous scales to assess overall image quality: “optimal” or “suboptimal” (23); “yes” or “no” to evaluate reflux into the extrahepatic bile duct in T1-weighted (T1W) sequences (21); “yes” or “no” with respect to T2W signal in the upper GI tract in general (17) and with respect to the presence of T2W signal overlapping and obscuring the common bile duct (CBD) and the pancreatic duct in particular (17).

Nine publications showed a significant reduction of GI fluid signal after administration of oral contrast agents (10,13–15,17,21,26,28,29). Few articles reported no significant changes in some parts of the upper GI tract (i.e. small intestine [especially the second portion of the duodenum], gastric body, or the gastric fundus) (17,21,26). One study showed that pineapple juice is inferior to ferumoxsil in its ability to suppress GI fluid signal (23).

Thirteen publications found a significant improvement of both overall image quality (10,21,22,26,32) and image quality of pancreatobiliary structures (9,11,14,15,21,22, 24,26,28–30). However, depending on the publication, depiction of some parts of the hepatobiliary and pancreatobiliary system on post-contrast MRCP was not significantly improved (11,13–15,17,21,26,28,29).

Seven publications assessed signal intensities in the GI tract, signal-to-noise ratios (SNR), contrast-to-noise ratios (CNR), and/or diameters of pancreatobiliary ducts quantitatively. After the intake of oral signal suppressors, the loss of gastric and duodenal signal was significant in comparison to the pre-contrast imaging (11,14,22,28). In general, SNR values of the pancreatobiliary ducts (in correlation to either air, abdominal fat, or liver) did not change significantly after the ingestion of oral signal suppressors (22,28,29), but decreased significantly after oral intake of MCT in particular (20). CNR values increased significantly between duodenum and/or stomach and pancreatobiliary ducts, respectively (14,22,29). Diameters of pancreatobiliary ducts showed no changes after the intake of oral signal suppressors (9).

Ten articles used additional diagnostic methods as a reference standard, e.g. ERCP (9,11,14,16,21,23,25,26, 30), computed tomography (CT) (16,21,26), surgery (9,11,25), MRCP follow-up (9,25), ultrasound (11,16), and esophagogastroduodenoscopy (19). Six publications proved high iron and manganese concentrations of the evaluated oral signal suppressors with spectroscopy (13–15,22,24,31).

Regarding the entirety of results and conclusions of the analyzed literature, there was an overall recommendation of the application of orally administered contrast agents in MRCP in 23 out of 25 publications. Advantages of using a signal suppressor include an improved visualization of the pancreatobiliary duct system compared to MRCP without the oral contrast agents (9–11,13–17,21,22,24,26,28–30). Furthermore, the use of signal suppressors assisted with differential diagnoses, such as fistulas between the duct system and the GI tract, ductal stones or duodenal diverticula (18,19,30). Two studies also emphasized the prevention of overlooking relevant diagnoses due to obscuring GI signal (9,30). Only one group stated that the use of the evaluated contrast material (ferrous gluconate; Lösferron®, Lilly Pharma, Bad Homburg, Germany) is not necessarily indicated as standard practice since there was no benefit compared to non-contrast MRCP images (25). One other publication warned of the loss of signal intensity in the CBD and reduced diagnostic image quality due to reflux of negative oral contrast agents in patients after sphincterotomy (27).

Fifteen of the 25 publications commented on taste and patient acceptance of oral signal suppressors. Substances like fruit juices and similar beverages (e.g. blueberry juice (12), acai juice (11,12), date syrup (13), black tea (28,29), pineapple juice (12,15,21,23,31), and also pineapple juice in combination with non-absorbable gadolinium-chelate (22,24)) were distinguished by good palatability and the absence of unwanted side effects. Publications on ferrous gluconate and gadopentetate dimeglumine also reported good patient tolerance, but did not provide any information on the subjective taste of the products (25,30). In contrast, three publications discussing ferumoxsil described limited patient acceptance and compliance due to bad taste and/or the required amount (9,23,31). One publication on carbon dioxide producing crystals mentioned reduced compliance due to an unfavorable odor of the product as well as hiccups as a side effect (17). Two groups reported mild and transient episodes of diarrhea after oral ingestion of ferric ammonium citrate (FAC; FerriSeltz®, Otsuka Pharmaceutical, Chiyoda, Tokyo, Japan) and MCT, respectively (10,26).

## Discussion

Standardized examination protocols for MRCP favor the use of oral contrast agents. Oral signal suppressors reduce superimposed fluid signal from the upper GI tract and hence improve the depiction of the pancreatobiliary duct system. In line with that, a majority of the publications reviewed in this study recommend the use of orally administered contrast agents in MRCP. Negative oral contrast agents not only improve the depiction of the pancreatobiliary duct system, but also aid with differential diagnoses (18,19,30) and prevent overlooking relevant pathology, especially in the pancreatic duct and CBD (9,30). However, some limitations in the use of oral contrast agents in MRCP were noted:

The contrast agent ferumoxsil, which is widely used and consists of nano-sized iron oxide crystals coated with siloxane, is known both for improvement of image quality in MRCP and displeasing, metallic taste (9,12,23,31). The latter is held responsible for reduced patient willingness to ingest the required amount of contrast material (9,23,31).

A common alternative to certified and/or pharmaceutically approved substances include iron- and manganese-rich fruit juices, which showed improved image quality in some studies. The advantage of good taste and palatability leads to an increased use in pediatric imaging (12,23). Some studies indicate no improvement of visualization of the pancreatic duct in comparison to MRCP without contrast (21) or differing levels of overall image quality depending on the iron or manganese content, type of juice, and manufacturer (12,15,31). Thus, a standardized recommendation is discouraged by some authors (12,15,31).

The negative contrast effect of oral agents used in MRCP is caused by shortening of the T2 relaxation time, which results in reduced signal intensities of fluids in the upper gastrointestinal tract on heavily T2W imaging. This effect is likely caused by paramagnetic effects in high concentrations of manganese and iron in oral signal suppressors. Signal loss in the biliary duct system greater than 28 min after ingestion of MCT (10 mg manganese/applied dosage) is attributed to the first pass effect of the manganese metabolism. Therefore, the authors suggest image acquisition swiftly after oral administration of the agent (20). Only three other authors in this review evaluated image quality and/or depiction of pancreatobiliary ducts at more than one time point after the administration of black tea or pineapple juice as signal suppressors, none of which discussed the influence of the manganese metabolism in particular. However, both Tang et al. and Ghanaati et al. detected no significance both in qualitative and quantitative assessment of the pancreatobiliary ductal system at two (5 and 15 min) or three (5, 10, and 15 min) time points after oral ingestion of black tea (28,29). Riordan et al. showed a significantly improved visualization of the pancreatobiliary duct structures 15 min after oral intake of pineapple juice compared pre-contrast imaging. However, they found no significant qualitative difference in diagnostic image quality for the ampulla, CBD, common hepatic duct, or intrahepatic ducts between pre-contrast and 30-min post-contrast MRCP. No quantitative assessment was done to confirm these findings. Whether an excretion of manganese, study design, chance, or an actual weakening effect of the oral contrast media (e.g. caused by dilution of the signal suppressor or aboral transport) are causative of these results remains unclear. Since the negative T2 contrast effect (level of suppressed fluid signal in the GI tract) was significantly better at 15 min and 30 min after the oral intake of pineapple juice, the latter appears improbable.

One of the few studies that did not recommend the use of the assessed substance in standard practice evaluated ferrous gluconate and observed no benefit in the visualization of the pancreatobiliary system compared to non-contrast MRCP (25). In contrast, a previous study evaluating the same agent in a smaller study group (10 versus 27 cases) recommended the use of ferrous gluconate. However, this smaller study merely evaluated the overall image quality of MRCP based on signal intensity of gastral and duodenal background signals; an assessment of the pancreatobiliary duct system was not included (32).

None of the reviewed studies found the use of negative oral contrast agents to be detrimental to MRCP image quality.

The majority of authors performed a subjective qualitative assessment of the obtained images, as well as an evaluation of taste and palatability of the studied signal suppressor. The minority of studies included an objective quantitative assessment or correlation with a subsequent diagnostic procedure (e.g. ECRP), which substantiated and underlined the respective study’s subjective findings.

Limitations of the presented literature review include a possible selection bias of the analyzed publications since only articles in full text and retrievable from PubMed were included. Furthermore, the choice of search terms influenced the findings. Case reports were included to cover as many contrast agents as possible used in the daily routine, and to present a wide range of application scenarios and possible pitfalls when using oral contrast agents in MRCP. A quantitative meta-analysis was not feasible due to the heterogeneity of study designs.

In conclusion, the use of oral contrast media in MRCP appears to be superior over examination protocols without oral contrast material. Based on the presented literature review, the ideal oral signal suppressor should be cost-efficient, palatable, and able to achieve sufficient GI signal suppression in MRCP.

## Supplementary Material

Supplementary material
