# Supplementary material for: Efficacy of oral contrast agents for upper gastrointestinal signal suppression in MRCP: A systematic review of the literature
Source: Acta Radiol Open. 2017 Aug 30;6(9):2058460117727315. doi: 10.1177/2058460117727315 (PMC5582660; doi:10.1177/2058460117727315)
Supplement: Supplementary material [file supplementary_material.pdf]

***Supplementary Table 1 – Results of the literature research concerning agents used in MRCP to alter signals induced by fluids in the upper gastrointestinal tract***

| **Name of Agent and Number of Scientific Publications dedicated to that Agent** | | **Included Comparison or Case Report** | |
|---|---|---|---|
| *Chemical Name of Agent (Trade Name, Company)* | *n* | *Compared Agent or Topic of Case Report* | *n* |
| **ferumoxsil** (Lumirem®, Guerbet, Villepinte, France / Gastromark®, Mallinckrodt, Inc., Dublin, Ireland) | 4 (3 (11,33,34) / 1 (25)) | pineapple juice (25,33) | 2 |
| | | ferrous gluconate + barium sulfate (34) | 1 |
| | | no agent (11) | 1 |
| **ferric ammonium citrate** (FAC; FerriSeltz®, Otsuka Pharmaceutical, Chiyoda, Tokyo, Japan) | 4 (12,18,28,29) | MCT + no agent (28) | 1 |
| | | no agent (12,18) | 2 |
| | | **CR:** MRCP after sphincterotomy (29) | 1 |
| **pineapple juice** | 4 (14,17,23,33) | ferumoxsil (33) | 1 |
| | | blueberry + acai juice (14) | 1 |
| | | ranitidine + no agent (23) | 1 |
| | | no agent (17) | 1 |
| **gadopentetate dimeglumine** (Gd-DTPA) | 2 (20,32) | no agent (20,32) | 2 |
| | | **CR:** duodenal diverticulum (20) | 1 |

| | | | |
|---|---|---|---|
| **manganese chloride tetrahydrate** (MCT; Bothdel Oral Solution®, Kyowa Hakko Kirin, Chiyoda, Tokyo, Japan / LumenHance®, Bracco Diagnostics, Milan, Italy) | 3 (21,22,28) | ferric ammonium citrate + no agent (28) | 1 |
| | | MCT (different time points after intake) (22) | 1 |
| | | **CR:** IPMN (21) | 1 |
| **ferrous gluconate** (Lösferron®, Lilly Pharma, Bad Homburg, Germany) | 2 (27,34) | ferumoxsil + barium sulfate (34) | 1 |
| | | no agent (27) | 1 |
| **blueberry juice** | 2 (14,16) | pineapple + acai juice (14) | 1 |
| | | no agent (16) | 1 |
| **acai juice** | 2 (13,14) | pineapple + blueberry juice (14) | 1 |
| | | no agent (13) | 1 |
| **black tea** | 2 (30,31) | no agent (30,31) | 2 |
| **date syrup** | 1 (15) | no agent (15) | 1 |
| **mixture of Gd-DOTA / pineapple juice** | 1 (24) | no agent (24) | 1 |
| **mixture of Gd-DTPA / pineapple juice** | 1 (26) | no agent (26) | 1 |
| **ferristene** (Abdoscan®, Nycomed SA, Oslo, Norway) | 1 (35) | | |
| **carbon dioxide producing crystals** | 1 (19) | no agent (19) | 1 |
| **barium sulfate** (Micropaque®, Guerbet, Villepinte, France) | 1 (34) | ferumoxsil + ferrous gluconate (34) | 1 |
| **ranitidine** | 1 (23) | pineapple juice + no agent (23) | 1 |

n – Number of Publications; CR – Case Report
